# Chromosome numbers of populations of three varieties of *Bidens pilosa* in Taiwan

**DOI:** 10.1186/s40529-015-0107-5

**Published:** 2015-09-18

**Authors:** Ya-Lun Huang, Wen-Yuan Kao

**Affiliations:** 1 Institute of Ecology and Evolutionary Biology, National Taiwan University, 1, Roosevelt Rd., Sec. 4, Taipei, 106 Taiwan; 2 Department of Life Science, National Taiwan University, 1, Roosevelt Rd., Sec. 4, Taipei, 106 Taiwan

**Keywords:** *Bidens pilosa*, Chromosome number, Crossing, Invasive plant, Self-incompatibility

## Abstract

**Background:**

Hairy beggar-ticks (*Bidens pilosa* L.) is a common invasive plant in tropical and subtropical regions. The Flora of Taiwan listed three varieties of *B. pilosa* in Taiwan, var. *minor*, var. *pilosa* and var. *radiata*. Among the three varieties, var. *radiata* was the most recently, in 1970s, introduced into Taiwan. However, after its introduction into Taiwan, var. *radiata* has become dominant over the other two varieties and is considered a serious invasive plant in lowland of Taiwan. Our previous study showed that var. *radiata* is self-incompatible and the other two varieties are self-fertile. Could it be possible that different chromosome numbers contribute to the different breeding systems of these three varieties? In addition, the heterogeneities of traits of var. *radiata* were found higher than those of var. *minor* and var. *pilosa*. Is the phenomenon resulting from the hybridization between var. *radiata* with other varieties? We counted chromosome numbers of populations of these three varieties distributed in Taiwan and conducted hand pollination treatment between var. *radiata* (as pollen receiver) and var. *minor* or var. *pilosa* (as pollen donor) to provide answer for the aforementioned questions.

**Results:**

No difference was found in chromosome numbers among populations of the same variety. Forty-eight chromosomes (2*n* = 48) were counted for var. *radiata* while 72 (2*n* = 72) chromosomes for var. *minor* and var. *pilosa.* Therefore, var. *radiata* is tetraploid and var. *minor* and var. *pilosa* are hexaploid. No successful hybridization was found between var. *radiata* and var. *minor* or between var. *radiata* and var. *pilosa*.

**Conclusions:**

This study provided the evidence that the invasive plant (*B. pilosa* var. *radiata*) has different chromosome numbers from the other two varieties and is unlikely to hybridize with the other two varieties.

## Background

**Fig. 1:**
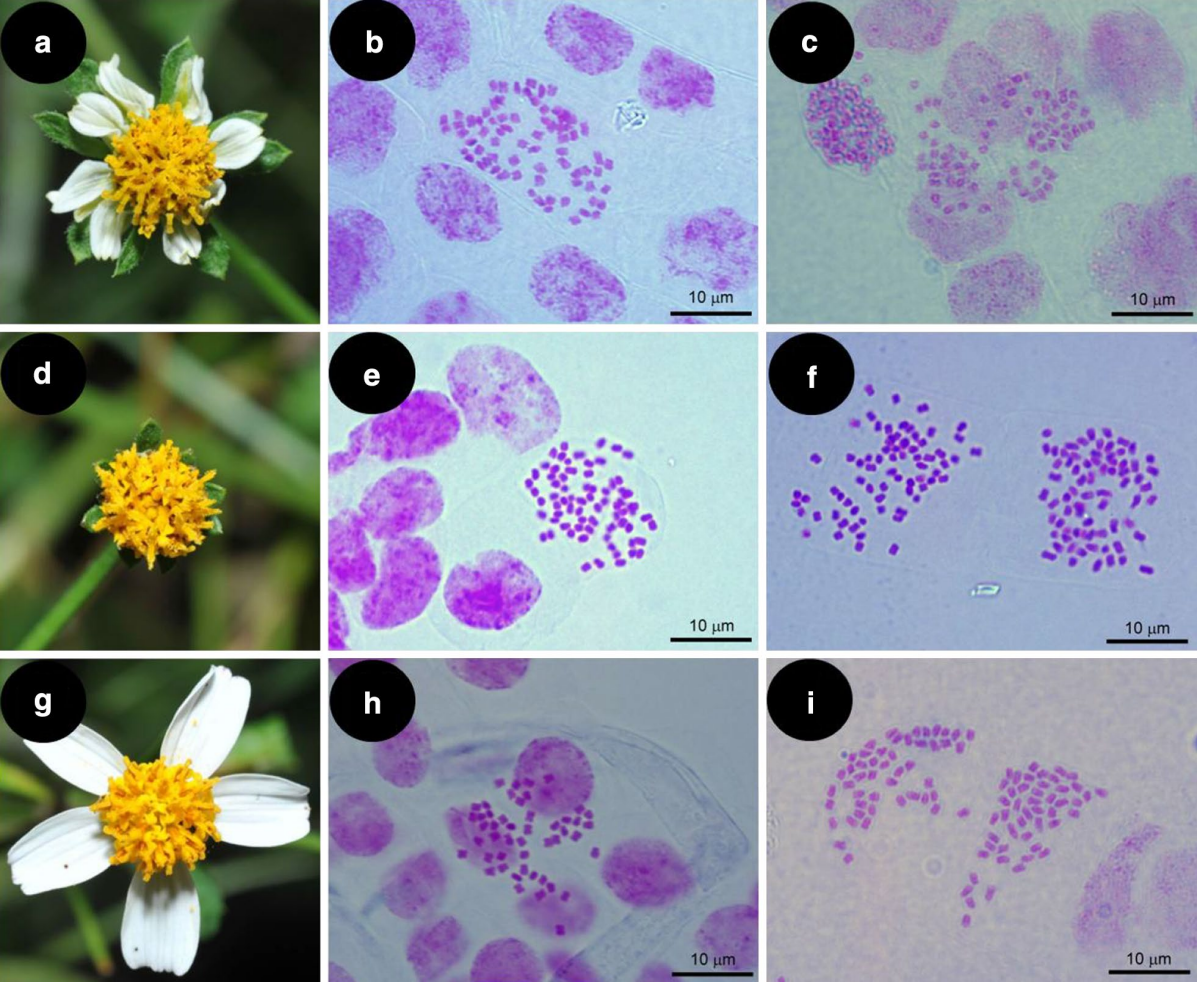
Pictures of inflorescence and somatic chromosomes of three varieties of *Bidens pilosa* in Taiwan. Representatives of inflorescence of var. *minor* (**a**), var. *pilosa* (**d**) and var. *radiata* (**g**) and somatic chromosomes of roots sampled from two different populations of each variety: var. *minor* from Chiayi (2*n* = 72) (**b**) and from Yilan (2*n* = 72) (**c**), var. *pilosa* from Hualien (2*n* = 72) (**e**) and from Chiayi (2*n* = 72) (**f**) and var. *radiata* from Nantou (2*n* = 48) (**h**) and from Yilan (2*n* = 48) (**i**) county in Taiwan

*Bidens pilosa* L. (Hairy beggar-ticks, Asteraceae) is a cosmopolitan weed. With its diversification center in Mexico, it is widely distributed in tropical and subtropical regions (Ballard 1986). This taxon is an annual or perennial herb with square stems and opposite leaves and often occupies roadsides, disturbed sites and cultivated fields (Peng et al. 1998). Based on the morphological differences, *B. pilosa* was classified into six varieties: var. *pilosa*, var. *minor*, var. *radiata*, var. *bimucronata*, var. *calcicola*, and var. *alausensis* (Sherff 1937). Accordingly, The Flora of Taiwan listed three varieties of *B. pilosa* L. in Taiwan, i.e. var. *minor*, var. *pilosa* and var. *radiata* (Peng et al. 1998). The most obvious differences in morphology among these three varieties are their flowers (Fig. 1). The capitula of var. *pilosa* are discoid (without ray florets), and the capitula of var. *minor* and var. *radiata* are radiate (with ray florets). Although var. *minor* and var. *radiata* both have ray florets, their ray florets are quite different. *B. pilosa* var. *minor* have eight ray florets which are often shorter than 8 mm while var. *radiata* have 5–8 ray florets which are often longer than 10 mm. Thirty years ago, var. *minor* was widely distributed in Taiwan. However, after var. *radiata* being introduced into Taiwan, var. *radiata* has become a serious invasive plant and dominant over the other two varieties within 30 years (Wu et al. 2004, 2010).

A previous study on breeding systems of these three varieties demonstrated that var. *minor* and var. *pilosa* are self-compatible while var. *radiata* is self-incompatible (Huang and Kao 2014). Chromosome numbers and breeding systems of 200 populations of *B. pilosa* in southern United States, Mexico and Central America have been investigated to clarify the classification of *B. pilosa* (Ballard 1986). After the analyses, Ballard concluded that *B. pilosa* in North and Central America should be treated as a species complex containing three species with different chromosome numbers due to different ploidy: *B. odorata* (2*n* = 24), *B. alba* (2*n* = 48) and *B. pilosa* (2*n* = 72). Species with different polyploidy also had different breeding system: hexaploid populations were self-fertile while diploid and tetraploid populations were self-incompatible. According to Ballard’s result, could it be possible that the chromosome number of var. *radiata* is different from that of var. *minor* and var. *pilosa*?

Furthermore, many traits of var. *radiata* were found having higher variation than those of var. *minor* and var. *pilosa*. For example, highly variation in ray floret numbers and sizes was found in var. *radiata*, while all var. *minor* had eight ray florets with similar size in each capitulum. Significant variations in pollen/ovule ratio and disc floret numbers per capitulum were only found in var. *radiata* but not in var. *minor* and var. *pilosa* (Huang and Kao 2014). Because var. *radiata* is self-incompatible and sometimes growing sympatrically with var. *minor* and var. *pilosa*, could it be possible that the var. *radiata* can hybridize with the other two varieties resulting in the highly variation in these traits?

The objectives of this study were to understand (1) whether these three varieties have different chromosome numbers, and (2) whether *B. pilosa* var. *radiata* can hybridize with var. *minor* or var. *pilosa*. To achieve the goal, we counted chromosome numbers of populations of these three varieties in Taiwan and conducted hand pollination treatments between var. *radiata* (as pollen receiver) and var. *minor* or var. *pilosa* (as pollen donors).

## Methods

### Chromosome count

**Fig. 2:**
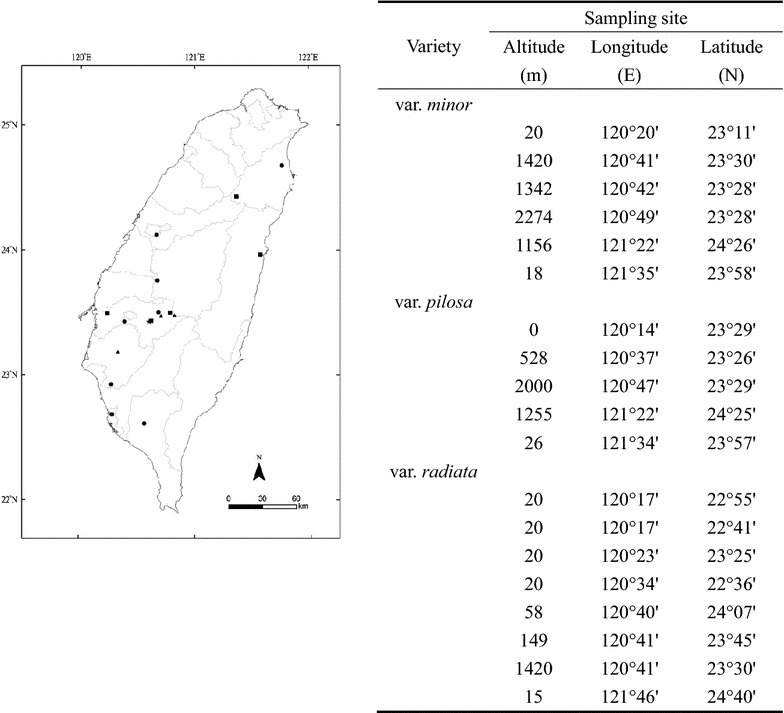
Sampling sites of the three varieties of *Bidens pilosa* in Taiwan. Achenes used in chromosome numbers experiment were collected from six populations of *B. pilosa* var. *minor* (*filled triangle*), five populations of var. *pilosa* (*filled square*), and eight populations of var. *radiata* (*filled circle*). The altitude, longitude and latitude of these 19 sampling sites were also listed. Achenes used in hand pollination experiment were collected from populations of the three varieties growing sympatrically in Chiayi (*filled star*) (120°37′E, 23°26′N, 500 m a.s.l.)

Achenes of three varieties of *B. pilosa* were collected from 5–8 populations distributed in Taiwan (Fig. 2). The collected achenes were planted in 1 L pots for about 1 month. When plants reached 20 cm height with 4–5 paired leaves, their shoots were cut and immersed into water for growing adventitious roots (Hsu and Kao 2014).

When shoots growing adventitious roots of 3–5 cm, the emergent rootlets with root-tips (ca. 1–2 cm) were cut, pretreated with 2 mM 8-hydroxyquinoline at 14 °C for 5 h, fixed in Carnoy’s solution (95 % EtOH: acetic acid = 3:1, v/v) for 24 h and then stored in 70 % EtOH at −20 °C. After washed with distilled water twice, rootlets were hydrolyzed in 1 N HCl at 60 °C for 8 min and rinsed twice with distilled water. Following, materials were stained with leuco-basic fuchsin in the dark at room temperature for 2 h and treated with 1 % pectinase at room temperature for 1 h to separate the connected cells. Root-tips were spread on a slide and squashed in a drop of 45 % acetic acid and then observed under a microscope (Grombone-Guaratini et al. 2006). Totally, 16 cells of var. *minor*, 7 cells of var. *pilosa* and 43 cells of var. *radiata* were counted.

### Hand-pollination experiment

Achenes collected from a population in Chiayi (120°37′E, 23°26′N, 500 m a.s.l.) (Fig. 2, filled star), where the three varieties grew sympatrically, were planted in pots and placed in the experimental farm of National Taiwan University. After about 2 months, when these plants bloomed, their flowers were used in crossing trials. Because var. *minor* and var. *pilosa* are highly self-compatible and capable of self-pollinating (Huang and Kao 2014), it is difficult to prevent their pollen grains depositing on their own stigma. Therefore, these two varieties were assigned as pollen donors in crossing trials. In contrast, var. *radiata* being self-incompatible (Huang and Kao 2014) was assigned as pollen receivers in crosses. In addition, crosses between individuals of var. *radiata* from different populations were also conducted as control. Plants growing naturally in the experimental farm of National Taiwan University and those plants growing from the achenes collected from the Chiayi population were used as pollen donors and receivers, respectively.

*Bidens pilosa* produces inflorescence of capitulum. In its capitulum, the hermaphroditic disc florets opened in centripetal sequence, and the duration from the blooming of the first disc floret to the last disc floret is about 3–4 days (Huang et al. 2012). Therefore, hand pollination was conducted once a day consecutively for 3 or 4 days during the period of anthesis. To prevent the deposition of non-target pollen grains, the capitula assigned as pollen receivers were wrapped in fine nylon-mesh netting bags (7 × 9 cm) as soon as their first disc florets opened until all disc florets on each capitulum withered. The capitula assigned as pollen donors were also wrapped into the netting bags to prevent contamination of pollen grains from other species.

At harvesting, the numbers of undeveloped ovules, immature and mature achenes were counted. Because there is only one ovule in each disc floret (Huang et al. 2012), the sum of mature achenes, immature achenes and undeveloped ovules represent the numbers of disc floret per capitulum.

## Results

### Chromosome count

The chromosomes of *B. pilosa* var. *radiata*, var. *minor* and var. *pilosa* shared similar size (ca. 2 μm in length) and morphology (with centromeres in the central region of the chromosomes) (Fig. 1).

For each variety, plants collected from different populations had same chromosome numbers. However, different chromosome numbers were counted among the three varieties. Chromosome numbers of var. *minor* and var. *pilosa* were counted as 2*n* = 72 (Fig. 1b, c, e and f), while those of var. *radaita* was 2*n* = 48 (Fig. 1h, i).

### Hand-pollination experiment

In the crossing experiment, the capitulum of *B. pilosa* var. *radiata* assigned as pollen receivers had 21–57 disc florets in each capitulum (Table 1). In crossing trial, no viable achenes were produced when the stigmata of disc florets of var. *radiata* receiving either the pollen grains from var. *minor* or var. *pilosa* (Table 1) or pollen grains of their own. In contrast, 17–29 mature achenes were produced in one capitulum of var. *radiata* when their stigmata receiving pollen grains from other individuals of var. *radiata*.

**Table 1:** Disc floret numbers and achene set in hand pollination experiment

Pollen donor	Pollen receiver (var. *radiata*)		
	Disc floret	Mature achene	*n*
var. *minor*	48.7 ± 3.4	0 ± 0	3
var. *pilosa*	38.8 ± 2.3	0 ± 0	5
var. *radaita* (self)	31.8 ± 4.5	0 ± 0	4
var. *radiata* (cross)	42.3 ± 3.3	23.0 ± 2.0	6

The disc floret number per capitulum, mature achene number per capitulum (mean ± SE), and the number of capitulum counted (*n*) in crossing experiment by hand pollination in which *Bidens pilosa* var. *radiata* as a pollen receiver, receiving pollen from other varieties (var. *minor* or var. *pilosa*) or same individual of var. *radiata* (self), or other individuals of var. *radiata* (cross)

## Discussion

Different chromosome numbers were found between the invasive and non-invasive varieties of *B. pilosa* in Taiwan. Different ploid level is one of the most frequent reasons leading to difference in chromosome numbers in some genera of Asteraceae (Dematteis and Fernandez 2000; Keil et al. 1988; Solbrig et al. 1972). Therefore, the variation in chromosome numbers among the three varieties of *B. pilosa* might result from Polyploidy. The basic chromosome number for genus *Bidens* has been reported as *x* = 12 (Ballard 1986). Accordingly, *B. pilosa* var. *radiata* is tetraploidy, while var. *minor* and var. *pilosa* are hexaploidy. A study on the breeding system of these three varieties found that var. *radiata* was self-incompatible, while var. *minor* and var. *pilosa* were self-compatible (Huang and Kao 2014). Thus, different ploid level might result in the different breeding systems among the three varieties. Similar results were also reported for 200 populations of *B. pilosa* species complex in Central America, where the tetraploid populations were self-incompatible while the hexaploid populations were self-compatible (Ballard 1986).

Recent studies showed that there is a link between Polyploidy and invasiveness (Beest et al. 2012; Goralski et al. 2014; Lowry and Lester 2006; Treier et al. 2009). A study compared 81 invasive species and their 2356 congeners found that invasive species are generally polyploidy (Pandit et al. 2011). In this study we found that the invasive var. *radiata* is polyploidy. However, the non-invasive varieties are also polyploidy and even have higher ploid level than the invasive one. In addition, *B. bipinnata*, the congener of the three varieties, which is not invasive in Taiwan is also a polyploidy (2*n* = 72) (Peng and Hsu 1978). Furthermore, it is reported that in the native distribution range of *B. pilosa* species complex, var. *minor* and var. *pilosa* are more widespread and more dominant than var. *radiata*. Thus, Polyploidy alone cannot explain why var. *radiata* becomes more dominant than the other two varieties in Taiwan. There are some other traits, for example, the ability of vegetative reproduction (Hsu and Kao 2014) and its breeding system (Huang and Kao 2014), contributing to the invasiveness of var. *radiata* in Taiwan.

Results from hand pollination experiment and consistent chromosome numbers counted in populations of var. *radiata* indicated that var. *radiata* is unlikely to hybridize with var. *minor* or var. *pilosa*. Accordingly, highly variation in traits found in *B. pilosa* var. *radiata* is unlikely caused by hybridizing with other varieties. Instead, we have previously suggested that the highly heterogeneity in traits might result from the obligate xenogamous breeding system carried by var. *radiata* (Huang and Kao 2014). The characters of highly variation in many traits might play an important role contributing to the invasiveness of var. *radiata* in Taiwan.

Based on the morphological differences, Sherff (1937) classified *B. pilosa* into six varieties. Ballard (1986) investigated 200 populations of *B. pilosa* in central America and suggested that plants previously classified as var. *pilosa* and var. *minor* by Sherff’s system still belonged to *B. pilosa* (hexaploid, 2*n* = 72). But those being classified as var. *radiata* by Sherff should be treated as *B. odorata* (diploid, 2*n* = 24) or *B. alba* (tetraploid, 2*n* = 48), depending on their chromosome numbers. Accordingly, *B. pilosa* var. *radiata* in Taiwan might be treated as *B. alba*. Further studies on detailed morphological characters, geographic distribution and molecular phylogeny of the genus *Bidens* are suggested to confirm the systematics of the three varieties in Taiwan.

## Conclusions

The invasive and non-invasive varieties of *Bidens pilosa* in Taiwan have different chromosome numbers and were different polyploid. The chromosome number of invasive var. *radiata* was 2*n* = 48 (tetraploidy), while those of non-invasive var. *minor* and var. *pilosa* were 2*n* = 72 (hexaploidy). The hand pollination experiment demonstrated that the invasive var. *radiata* is unlikely to hybridize with the non-invasive var. *minor* or var. *pilosa*. High variance of many traits found in invasive var. *radiata* might result from its self-incompatibility.
